# Contrasting Roles of Cannabidiol as an Insecticide and Rescuing Agent for Ethanol–induced Death in the Tobacco Hornworm *Manduca sexta*

**DOI:** 10.1038/s41598-019-47017-7

**Published:** 2019-07-19

**Authors:** Sang-Hyuck Park, S. Kyle Staples, Eric L. Gostin, Jeffrey P. Smith, Jose J. Vigil, Dustin Seifried, Chad Kinney, Christopher S. Pauli, Brian D. Vanden Heuvel

**Affiliations:** 1 Department of Biology, Colorado State University-Pueblo, Pueblo, CO 81001 USA; 2 Institute of Cannabis Research, Colorado State University-Pueblo, Pueblo, CO 81001 USA; 3 Department of Chemistry, Colorado State University-Pueblo, Pueblo, CO 81001 USA

**Keywords:** Plant physiology, Spinal cord, Drug development

## Abstract

*Cannabis sativa*, also known as marijuana or hemp, produces a non-psychoactive compound cannabidiol (CBD). To investigate the defensive role of CBD, a feeding preference assay was performed with tobacco hornworm *Manduca sexta*. The larvae clearly show feeding preference towards the *Cannabis* tissue containing low CBD over high CBD. While the larva avoided the high CBD diet, we investigated detrimental effects of CBD in the insects’ diet. Contrasted to the performance on low CBD-infused artificial diet (AD), larvae reared on the high CBD diet suffer significantly reduced growth and increased mortality. Through testing different carriers, we found that the increase of EtOH in the diet is negatively correlated with insect development and behaviors. Notably, CBD treatment significantly improved ethanol-intoxicated larval survival rate by 40% and also improved diet searching activity, resulting in increased diet consumption. Electrophysiology results revealed that the CBD-treated ganglia had delayed but much larger response with electric stimuli in comparison to the larvae reared on AD only and EtOH-added diet. Our results show CBDs’ defensive role against pest insects, which suggests its possible use as an insecticide. We also provide evidence that CBD alleviates alcohol-induced stress; consequently, improving the performance and viability of *M*. *sexta* larvae.

## Introduction

*Cannabis*, is primarily an annual dioecious plant that produces secondary metabolites called cannabinoids that are mainly accumulated in the glandular trichomes of the plant^1^. *Cannabis* produces at least 120 different cannabinoids that are C_21_ terpenophenolic compounds, which synthesized from olivetolic acid and geranyl diphosphate (GPP)^2^. These chemical constituents, with the aid of geranyltransferase lead to the formation of the central precursor cannabigerolic acid (CBGA), which allows for the conversion to cannabidiolic acid (CBDA) and tetrahyrdocannabinolic acid (THCA)^3^. These two acidic forms of cannabinoids undergo decarboxylation, converting into cannabidiol (CBD) and (−)-Δ9-trans-tetrahydrocannabinol (Δ9 –THC) by CBDA and THCA synthase, respectively^4^. Cannabidiol and CBDA are the dominant constituents of non-psychotropic (hemp-type) *C*. *sativa* L. Hemp-type *Cannabis* can produce larger amounts of CBDA, sometimes to levels that exceed 40% of the cannabinoid extract from the whole plant^1^.

Hemp and other cannabinoid containing varieties have been touted for their insecticidal properties^5,6^. Although very little is known about the mechanism cannabinoids use to protect against harmful pest insects and microbial pathogens, several studies revealed that aqueous and solvent extracts of *Cannabis*, specifically hemp extracts, effectively repel insects and inhibit the growth of soil-borne microbial pathogens^5,6^. Several farmers and agricultural researchers have reported that rotating fields between crops with hemp or planting it along the perimeter of fields reduces insect infestations^6^. Despite the merit of *Cannabis* extract as a biocontrol agent, it is not currently understood which constituents of hemp confer the insecticidal function, the inhibitory mechanisms, and possible synergistic interactions with other metabolites (*e*.*g*., terpenoid aldehydes) in the defense mechanisms.

In addition to the agronomic value of hemp, over the past decades, CBD has been studied for therapeutic potential as an anti-inflammatory^7^, antioxydant^8^, neuroprotectant^9^, anticonvulsant^10^, anti-panic^11^, anxiolytic^12^, antidepressant^13^, analgesic^14^, anti-tumoral agent^15^, and anti-psychotic^16^. Recently, more attention has been given toward CBD’s neuro-therapeutic effects on patients suffering from autism and chronic seizures of epilepsy^17,18^. To investigate the cause of the observed beneficial effects in mice with epilepsy and autistic-like behaviors, one group correlated CBD use with increased inhibitory postsynaptic potentials and decreased excitatory postsynaptic potential in response to strong stimuli^18^.

In addition, a study indicated the protective effects of CBD on mice livers by possibly preventing the increase of free radical and oxidative stress when the subjects were intoxicated with ethanol^19^. Other studies demonstrated CBD’s efficacy as an *in vivo* neuroprotectant to prevent binge ethanol-induced brain injury in rats^20^, and that CBD reduced ethanol consumption, motivation and relapse in mice^21^. These studies suggest that CBD lessens the ethanol-induced oxidative stresses. Similar CBD studies performed in human showed that CBD lowered blood ethanol levels; although, no clear improvement on motor and mental performance were shown^22,23^.

In this research, we examined if CBD has a defensive role in *Cannabis* plants against the pest insect *Manduca sexta*. The feeding preference assays demonstrated that CBD acts as the deterrent property of hemp plants, which furthers our understanding of the ecological and agricultural benefits of cannabinoid production. Additionally, we observed an unexpected rescuing effect of CBD in the ethanol-induced hornworm death, which we further investigated through electrophysiology. This study aimed to examine these dual, contrasting roles of CBD as an insecticide, as well as a potential therapeutic agent for alcoholism/addiction treatments.

## Methods

### Hornworm preparation and cannabidiol treatment

In this study, *M*. *sexta* was used as the pest insect in the feeding assays because of several advantages over others, including its large size (100 mm long and 10 grams), short life cycle (30–50 days), and ease of rearing under laboratory conditions. *M*. *sexta* eggs were obtained from Carolina Biological Supply (Burlington, NC). The larvae were individually placed in a round plastic container (100 mm × 50 mm) with a perforated lid. For feeding studies, 1^st^ instar larvae were reared on 40 g of AD at 25 °C on a 12:12 light-dark cycle^24^. Cannabidiol stock solution (200 mM) was prepared in 20 ml of EtOH (200 proof) or medium chain triglyceride (MCT) oil (0.1%) by adding 1.26 g of >98% pure CBD isolate (Lilu’s Garden, CO). The stock solution was added to the AD to bring the final concentrations of the diets to10 μM, 100 μM, 1 mM and 2 mM CBD.

### *M*. *sexta* larval growth, mortality, and behavior measurements

Larval growth (*i*.*e*., length and weight) and mortality were monitored at 2-day intervals after being transferred to individual container until pupation. To examine if CBD affects larval behaviors, diet consumption and mobility were also monitored. The diet consumption was measured by weighing the diet loss of the container between 1^st^ instar larvae and pupation. All mobility experiments were performed using an automated, computerized system (Coulbourn Instruments, Holliston, MA). The system consisted of two Plexiglas chambers (203 mm × 228 mm × 254 mm), each equipped with a top-mounted 60 frame per second USB camera, and a LED light mounted on the side wall. Each chamber was placed inside a sound-attenuating isolation cubicle, which was equipped with a ventilation fan that produced 60 dB white noise. The mobility response of 5^th^-instar *M*. *sexta* larvae was captured at 60 frames per second for 10 mins., and the video was analyzed using motion detection software (Actimetrics) to generate a motion index. For multiple group statistical comparisons, the differences of larval growth (*i*.*e*., size and weight) and motion index were analyzed by one-way ANOVA with Tukey’s post-test. The log-rank (Mantel-Cox) test was used for survival curve comparisons. All the statistical analyses were performed using the GraphPad Prism software package (GraphPad Software, Inc., La Jolla, CA).

### CBD quantification in *M*. *sexta* larval dry weight via high pressure liquid chromatograph (HPLC)

The CBD extraction from *M*. *sexta* larval dry weight was conducted using a modified United Nations Office on Drugs and Crime (UNODC) method for phytochemicals from *Cannabis*^25^. Frozen larval samples were suspended with 5 ml of methanol (MeOH): chloroform solution (9:1, v/v) and then macerated with a clean glass stir rod. The macerated mixture was vortexed before being placed in a sonication, Bransonic 3510 (Branson Ultrasonics, Danburry CT) bath for 15 mins; vortexing every five mins. The sample was then centrifuged at 14,000 rpm for 15 mins and the supernatant was stored in a refrigerator at 4 °C until analysis. CBD contents were analyzed by ultra-high pressure liquid chromatograph (UHPLC) on a Dionex UltiMate 3000 (Fisher Scientific Company LLC, Pittsburgh, PA). Chromatographic separation of CBD was accomplished using an Accucore aQ C18 Polar Endcapped column (100 mm × 2.1 mm, particle size 2.6 µm) (Fisher Scientific Company LLC, Pittsburgh, PA). A stock solution of 100 μg/ml CBD diluted in MeOH was used to create the calibration curve, with 10 μg/ml, 50 μg/ml and 100 μg/ml being points on the curve. UHPLC analysis included external calibration with each set of samples, solvent blanks, and analysis of independently prepared continuing calibration verification (CCV) samples every twentieth sample to ensure accuracy. A binary gradient at a flow rate of 0.450 ml/min was used for separation. Mobile phase A was a 0.1% formic acid aqueous solution (Nanopure water, 18MΩ-cm) and mobile phase B consisted of MeOH. Cannabidiol was quantified by absorbance at 210 nm. The initial condition of 67% B with a non-linear gradient to 81% B from 0 to 8 mins gave the gradient a concave inflection point near 5 mins. The remaining gradients were linear. Mobile phase B was increased to 83% at 13 mins, then increased to 95% until 16 mins, and returned to 67% for 7 mins of equilibration. The differences in CBD accumulations were analyzed for statistical significance by one-way ANOVA with Tukey’s post-test available in the GraphPad Prism software package (GraphPad Software, Inc., La Jolla, CA).

### Electrophysiology on *M*. *sexta* larval ventral ganglion

The abdominal ganglion of 3^rd^ instar larva were dissected in hemolymph-like physiological saline (HL-3) buffer (70 mM NaCl, 5 mM KCl, 20 mM MgCl_2_·6H2O, 10 mM NaHCO_3_, 115 mM Sucrose, 5 mM trehalose, 5 mM HEPES, and 1.8 mM CaCl_2_, pH 7.2) to maintain the longevity of larval specimen for physiological recording^26^. The longitudinal mid-dorsal incision was performed and abdominal ganglia were dissected out of the larva. One end of segmental ganglion was pinned down in the recording chamber with nerve tubes attached at both ends. To measure the electric response of each nerve bundle, a bipolar concentric stimulating electrode (200 μm in diameter; FHC CBAEC75) was placed onto the ganglion, and a fire-polished low resistance borosilicate glass recording electrode, pulled with a PC-100 puller (Narishige Inc., Amityville, NY), was filled with HL3 solution and light suction was applied to draw the nerve bundle and hemolymph solution into the electrode for recording. The ganglia were stimulated with a square 0.1 ms duration pulse every 30 sec at increasing stimulation intensities controlled by a Stimulus Isolation Unit (Model BJN8–9V1) (Getting Instruments Inc., San Diego, CA). The responses were amplified with an Axoclamp-2A amplifier and recorded with pCLAMP 6 software (Axon Instruments, Union city, CA). Recordings were analyzed by Clampfit 6 (Axon Instruments, Union city, CA). The differences in electric response were analyzed for statistical significance by one-way ANOVA with Tukey’s post-test, which is available in the GraphPad Prism software package (GraphPad Software, Inc., La Jolla, CA).

## Results

### CBD as a potent feeding deterrent for *M*. *sexta*

**Figure 1:**
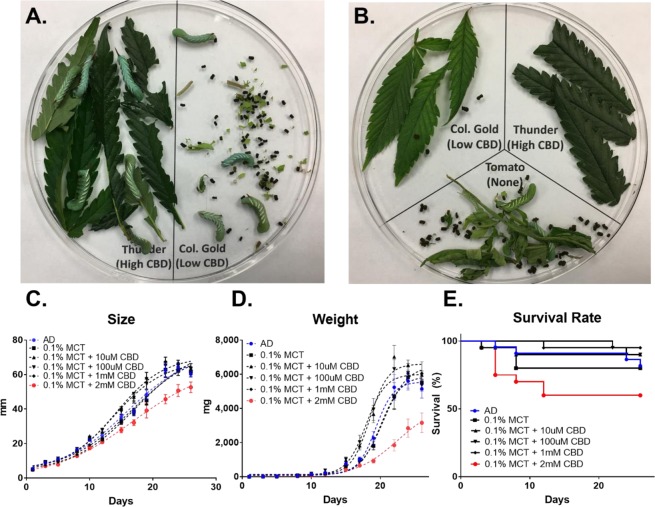
CBD as a potent feeding deterrent for *M*. *sexta*. (**A**) Two-way feeding preference test on the high CBD (3.2%) - and low CBD (0.81%)-producing *C*. *sativa* leaves. (**B**) Three-way feeding test with *Solanum lycopersicum* leaves. The size (**C**), weight (**D**), and mortality (**E**) of *M*. *sexta* when fed on AD containing 10 μM, 100 μM, 1 mM, and 2 mM CBD. For statistical analyses on insect growth and survival rate, a one-way ANOVA with Tukey’s multiple comparisons test (n = 20~22, *p* < 0.05) and Mantel-Cox test (n = 20~22, *p* < 0.05) were used, respectively.

To determine whether CBD plays a role against pest insects, 3^rd^ instar larvae were caged overnight in the dishes with equal amounts of *C*. *sativa* leaf tissues containing 3.2% CBD and 0.8% CBD, respectively (see Supplementary Table S1). The early stage larvae, *i*.*e*., neonates and 1^st^ instar, scraped the surface of leaves, but they were not able to consume the *C*. *sativa* leaf tissue, resulting in death. However, the juvenile 3^rd^ instar larvae had a measurable liking for the leaves containing lower CBD while showing avoidance of tissues containing high CBD (Fig. 1A). Three-way feeding preference test showed the same tendency that the larvae preferred no-CBD containing tomato or lower CBD tissues (Fig. 1B). After complete consumption of tomato leaves, the larvae moved to low CBD tissues but rarely consumed the high CBD tissues. This preliminary observation led us to test if CBD was a causal effect driving the feeding choice.

The larvae were raised on an artificial diet (AD) that is a soft wheat germ-based mixture. As a CBD solvent agent, medium-chain- triglyceride (MCT) oils were used because the oils are not known as a nerve-depressant, and show minimal effects on the larval growth and development under 0.1% MCT concentrations (see Supplementary Fig. S1). Figure 1C shows that 0.1% MCT oil readily dissolves varying amounts of CBD, ranging from 10 μM to 2 mM. Less than 1 mM CBD in AD did not affect larvae growth (black lines in Fig. 1C,D) or survival rate (black lines in Fig. 1E). At day 24, the average size of the larvae fed AD was 63.9 mm which was similar to larvae reared on 10 μM, 100 μM, and 1 mM, with average sizes of 62.1 mm, 65.1 mm, and 63.6 mm, respectively (n = 20~22). However, the size of larvae reared on AD containing 2 mM CBD was 50.7 mm, which was ~20% smaller than the larvae grown on 10 μM CBD (red line in Fig. 1C). At day 24, the average weight of larva reared on AD supplemented with 10 μM CBD was 6.5 g, which was 2.2-times greater than those of larvae reared on AD with 2 mM CBD (n = 12–16, *p* < 0.00001). Notably, the high dose of CBD (2 mM) significantly reduced the larval survival rate by 60%, while the lower dose of CBD at 10 μM, 100 μM, and 1 mM in AD shows at least 80% survival rates (Fig. 1E). It can be concluded that the high dose of CBD (2 mM) in the diet is detrimental to larval development and correlates to increased mortality, while less than 1 mM CBD concentration has no effect on development or mortality.

### CBD protects against ethanol toxicity in *M*. *sexta* larvae

**Figure 2:**
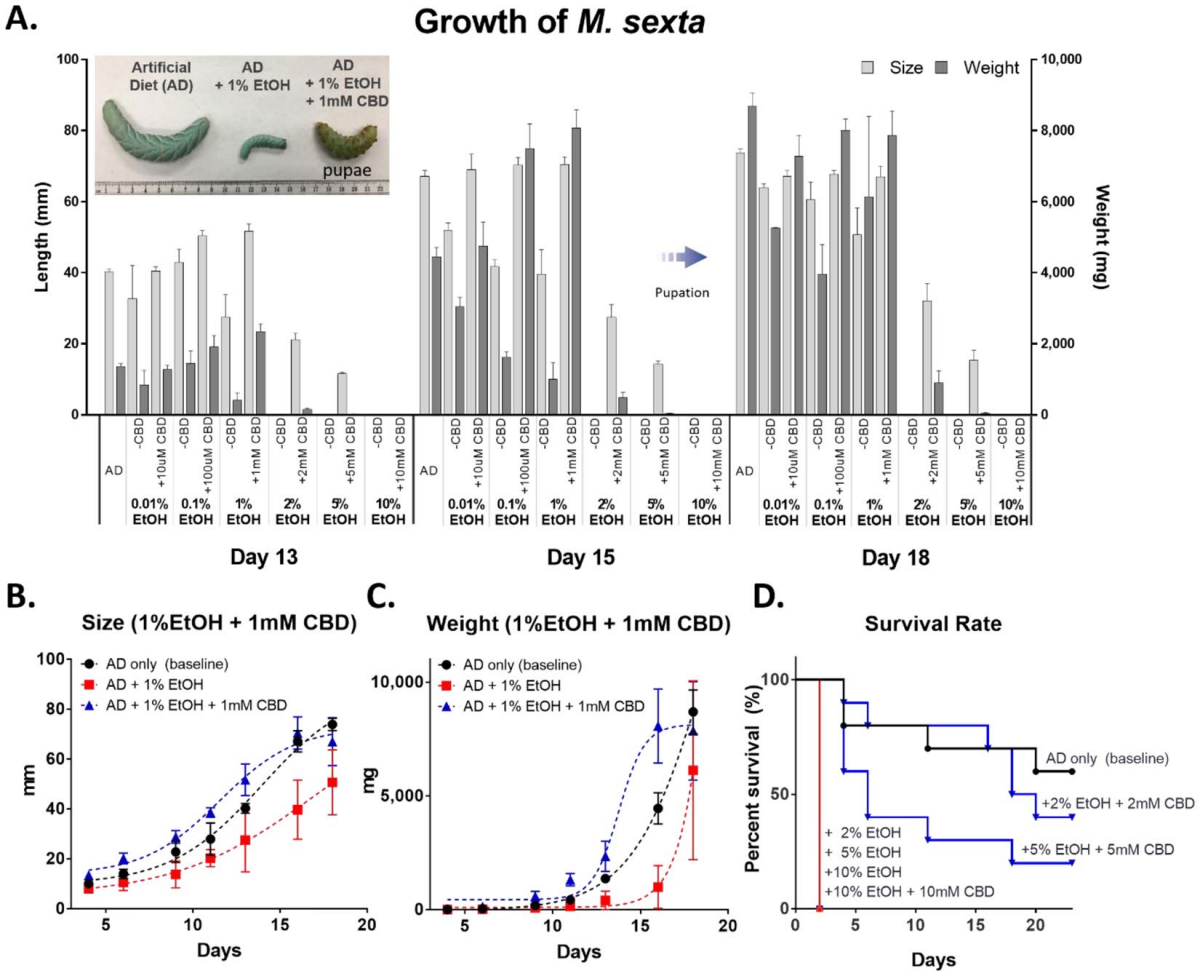
The effects of CBD on the growth and survival of *M*. *sexta* with EtOH solvent agent. (**A**) The size and weight increment between days 13 and 18. The inset in the upper left corner displays the size difference between larvae when fed different diets. Size (**B**) and weight (**C**) of insects reared on AD, AD + 1% EtOH, and AD + 1% EtOH + 1 mM CBD (one-way ANOVA with Tukey’s multiple comparisons test, n = 3~10, *p* < 0.05). (**D**) Survival rate of *M*. *sexta* larvae grown at 2%, 5%, and 10% of EtOH ± 2 mM, 5 mM, and 10 mM CBD concentrations (Mantel-Cox test, n = 5~10, *p* < 0.05).

Another commonly used CBD solvent agent, ethanol (EtOH, 200 proof), was tested to examine if there are any carrier effects of the MCT oil in the previous feeding study. 1^st^ instar larvae were incubated in AD, treated with varying EtOH concentrations (0–10%), with different amounts of CBD (10 μM to 10 mM). Figure 2A displays the growth of *M*. *sexta* larvae and the most active growth was observed during days 13 to 18. Although the growth of larvae treated with 1% EtOH were retarded, when compensated with 1 mM CBD, their growth returned to the level of the larvae reared on normal AD. The 1 mM CBD fed larva entered pupation earlier than those reared on normal AD; although, the differences were not statistically significant (*p* > 0.05).

Figure 2B,C represent the selective growth (*i*.*e*. length and weight) of *M*. *sexta* larvae reared on three different media; AD, AD + 1% EtOH, and AD + 1% EtOH + 1 mM CBD during days 16 to 18. Notably, the growth of the larvae reared on AD only (black line) and AD + 1% EtOH + 1 mM CBD (blue line) outperformed those reared on AD + 1% EtOH (red line) over a 23-day feeding period. The largest growth difference was shown on day 16. The average size of larvae reared on AD + 1% EtOH was 39.7 mm (n = 3); whereas, the larvae reared on AD + 1% EtOH + 1 mM CBD was 70.5 mm (n = 10, *p* < 0.05). In addition, the CBD-treated larvae showed a greater weight increase during the same time period. On day 16, the average weight of larvae reared on AD + 1% EtOH was 996.5 mg (n = 3) whereas the larvae on AD + 1% EtOH + 1 mM CBD was 8,072.5 mg (n = 10). The larval weight reared on CBD-treated AD was 8.1-times greater than the larvae reared on 1% EtOH-treated AD. On the other hand, no growth difference was found between the larvae reared on AD only and AD + 1% EtOH + 1 mM CBD (n = 7–10, *p* > 0.05). The insect feeding study showed that the increased levels of EtOH in the diets was negatively correlated with the larval growth and survival rate; however, the detrimental effects of EtOH were reduced when the larvae were treated with CBD.

Figure 2D represents the *M*. *sexta* larval survival rates. Greater than 1% EtOH in the diets was lethal all to *M*. *sexta* larvae. All larvae reared on 2%, 5%, and 10% EtOH concentrations died within three days, showing 100% mortality. However, the larvae that were administered CBD performed differently. The larvae reared on 2% EtOH + 2 mM CBD and 5% EtOH + 5 mM CBD, had increased survival rates of 40% and 20%, respectively. The larvae reared on 10% EtOH died regardless of the concentration of CBD administered.

### CBD increases food searching activity and diet consumption in *M*. *sexta* larvae

**Figure 3:**
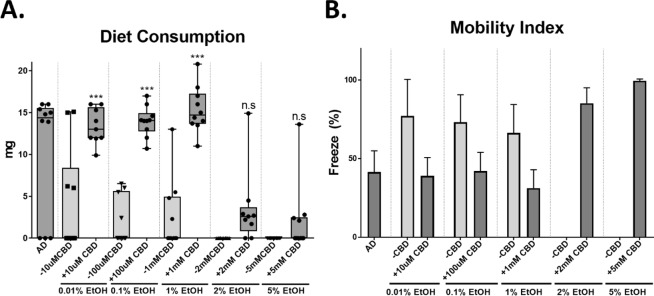
The effects of CBD on insect behaviors. (**A**) Diet consumption of insects reared on AD, AD + 0.01–5% EtOH, and AD + 0.01% EtOH + 10μM-5mMCBD (one-way ANOVA, Tukey’s multiple comparison at *p* < 0.05). (**B**) Insect mobility. The mobility is depicted as freeze %.

To examine if CBD affects insect behaviors under the three feeding conditions (AD, AD + 1% EtOH, and AD + 1% EtOH + 1 mM CBD), diet consumption and mobility of *M*. *sexta* larvae were measured. The diet consumption rate was recorded by measuring the total loss of diet starting from the first day of being transferred into the container until pupation. Figure 3A illustrates that CBD-fed *M*. *sexta* larvae consumed at least 3.1-times greater diets in comparison with those reared on EtOH-added to AD. However, the diet consumption of the survivors in 2 mM and 5 mM CBD-added AD was not significantly different than those of larvae reared on EtOH only treated diets (*p* > 0.05).

Additionally, larval mobility was recorded and the mobile index was depicted as the percentage (%) of freeze. Figure 3B compares the mobility index of the *M*. *sexta* treatment groups. The results show that 0.01–1% EtOH-treated larvae became less mobile (>70% freeze) compared to the larvae reared on AD only (<40% freeze). The high EtOH treatments (>2%) were lethal to hornworms; thus, no mobility index was recorded. Despite the addition of the high dose of CBD (2 mM and 5 mM) into AD containing 2% and 5% EtOH, respectively, the mobility remained low (80–100 freeze%).

### CBD rescues *M*. *sexta* larvae from EtOH-stress

**Figure 4:**
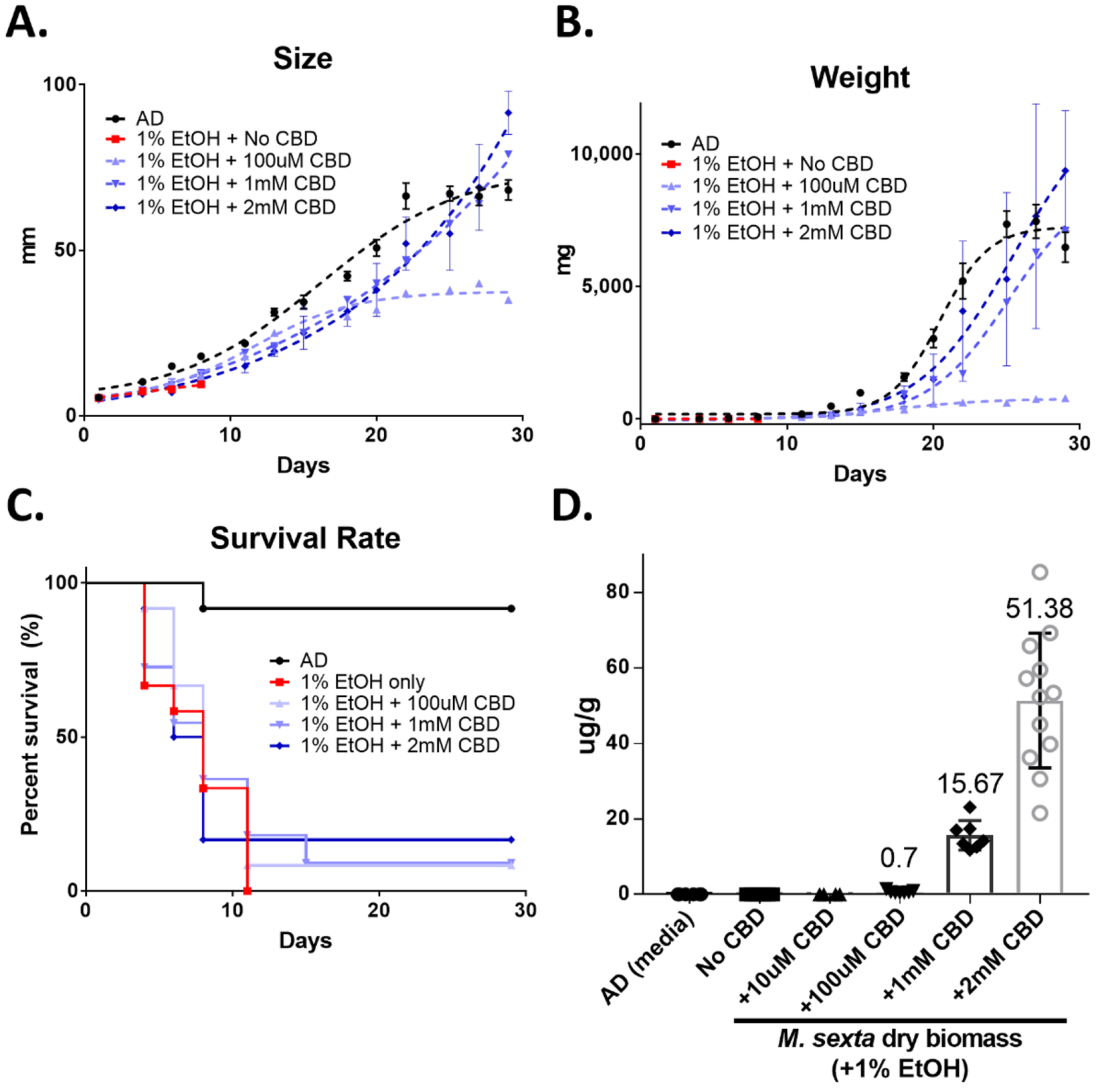
The growth and survival rate of *M*. *sexta* larvae reared on AD containing 1% EtOH with different CBD concentrations, ranging from 100 μM to 2 mM CBD. Comparison of size (**A**), weight (**B**), and survival rate (**C**) of larvae are shown. The larval survival rate was analyzed by Mantel-Cox test (n = 11~12, *p* < 0.05). (**D**) CBD accumulation in *M*. *sexta* larvae (μg of CBD/g of dry weight) when fed on different media. AD; artificial diet as negative control.

The original feeding study was performed in a semi-controlled greenhouse environment. To reaffirm the rescue effects of CBD, another feeding assay was performed in a controlled environment at 24 °C with a 12-h dark-light cycle. Figure 4 displays the growth pattern of *M*. *sexta* larvae reared on AD containing 1% EtOH with varying amounts of CBD (100 μM, 1 mM, and 2 mM). As shown previously, the larvae reared on AD + 1% EtOH showed 100% mortality within 10 days (red line in Fig. 4). Notably, the 2 mM CBD-administered larvae show normal growth patterns as seen in the larvae reared on AD, AD + 100 μM, and AD + 1 mM CBD. The larvae reared on 10 μM CBD show retarded growth, meaning that the low dose of CBD treatment did not rescue the EtOH-fed larva. Figure 4C represents the survival rate of *M*. *sexta* larvae reared on different CBD treatments. The larvae in the 100 μM, 1 mM and 2 mM CBD treatment groups exhibit increased survival rates of 8.3%, 9%, and 16.6%, respectively (Mantel-Cox test, n = 11~12, *p* < 0.05).

Figure 4D proves the accumulation of CBD in *M*. *sexta* larvae (μg of CBD/g of dry weight). As expected, AD media used as negative control, and the larvae reared on AD (depicted as No CBD) showed no CBD detection as well as the larvae reared on AD + 10 μM CBD. On the other hand, the larvae reared on AD + 100 μM CBD, + 1 mM CBD, and + 2 mM CBD accumulated positively correlated amounts of CBD in their body.

### CBD stimulates electrophysiological responses in the ventral chain ganglia

**Figure 5:**
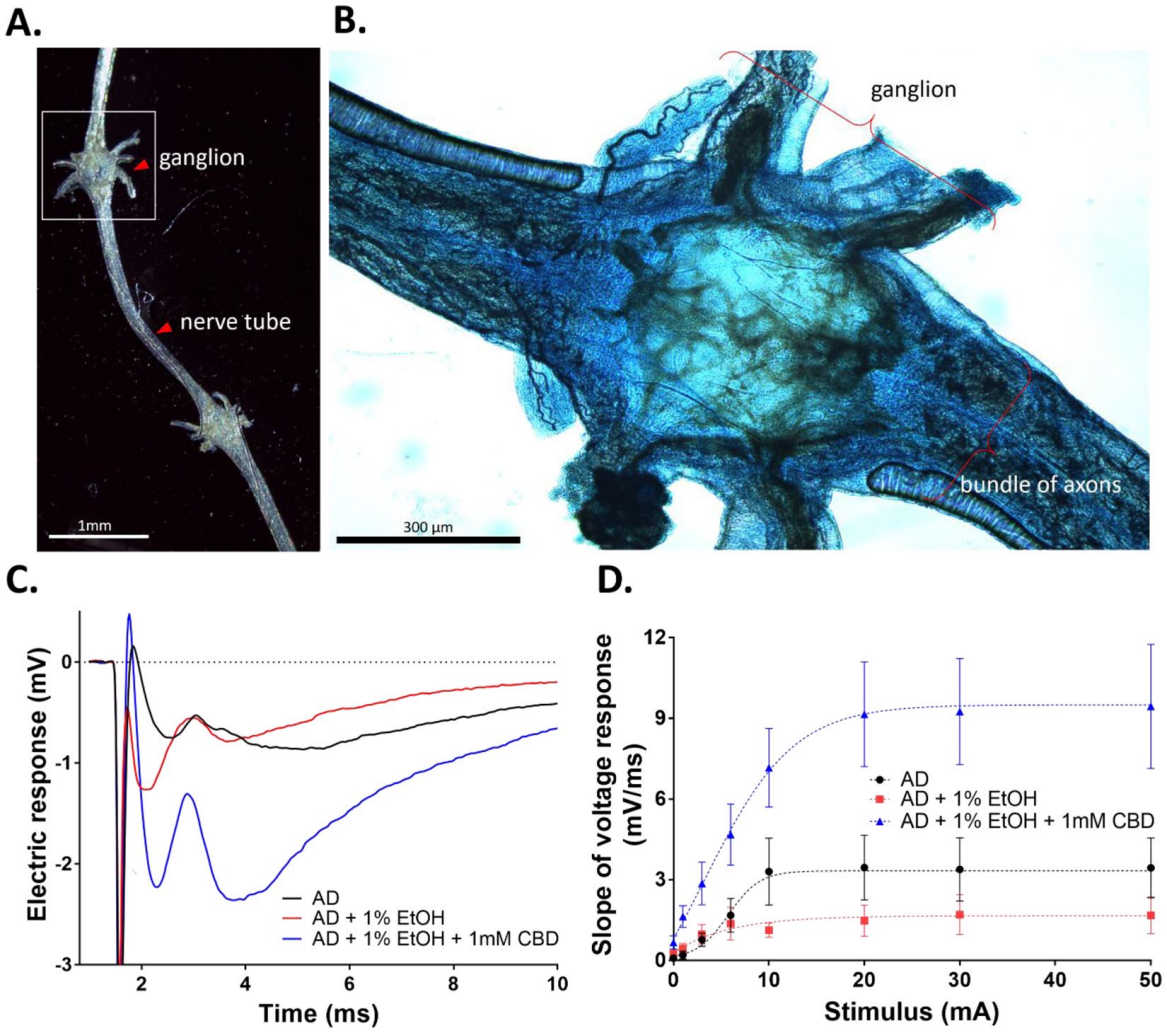
CBD increases electrophysiological responses measured in the ventral chain ganglia of *M*. *sexta* larvae. (**A**) A low power microscopic image of a 5^th^-instar chain ganglion which was micro dissected from the larva, and annotation showing where the stimulating electrode was placed in the ganglion, and where the nerve tube was cut and drawn into the recording electrode. (**B**) A high power image of a methylene blue-stained ganglion. (**C**) Representative voltage responses following electrical stimulation of ganglia from the *M*. *sexta* fed on AD, AD + 1% EtOH, and AD + 1% EtOH + 1 mM CBD (one-way ANOVA, n = 5, *p* < 0.05). (**D**) The slope of the negative voltage response plotted as a function the stimulus intensity to quantify the input-output relationships within each treatment group (one-way ANOVA, n = 5, *p* < 0.05).

To determine whether CBD affects the *M*. *sexta*’s nervous system, ventral chain ganglia were electrically stimulated and the voltage responses were recorded from the attached axons. Figure 5A,B show examples of the dissected larval ganglia and the positions of the recording and stimulating electrodes.

A stimulating electrode was placed into the middle of each ganglion, and the voltage responses were recorded at the cut end of the attached nerve bundles using a suction electrode. Figure 5C shows example voltage responses (mV/ms) recorded from the *M*. *sexta* larvae reared on AD, AD + 1% EtOH, and AD + 1% EtOH + 1 mM CBD. The electric responses appeared to be significantly increased in larvae fed AD + 1% EtOH + 1 mM CBD in comparison to the larvae fed either 1% EtOH containing AD or AD only. Responses from replicate larvae in each treatment group were quantified by calculating slope of the initial voltage response after stimulus artifact (1.28–1.76 ms), and the slope value (mV/ms) was plotted as a function of the input stimulus intensity. An input-output curve was then generated for each nerve preparation by applying incrementally increasing stimuli over a range of intensities (0–20 mA) and recording the integrated responses (Fig. 5D). The superimposed input-output plots show that the larvae reared on AD + 1% EtOH did not differ much from AD controls; whereas, the larvae that had been fed AD + 1% EtOH + 1 mM CBD showed more intense responses over the entire range of stimuli provided.

## Discussion

In *Cannabis*, two phytochemicals, THC and CBD, are abundantly produced in the glandular trichomes, and are expected to play pivotal defensive roles. Indeed, these cannabinoids are known to confer resistance against a variety of bacteria^27^. Also, several anecdotes show that high THC and CBD producing *Cannabis* varieties are more protective against microbial and insect pathogens. A recent study suggests the possible use of the crop-residue of fiber hemp containing CBD as an insecticide^5^. Despite the escalating pharmaceutical uses of CBD, the intrinsic questions of why *Cannabis* plants produce the secondary metabolites, how they exploit the molecules for their survival, and what evolutionary benefits they provide still remain unanswered.

To investigate which constituents of hemp confer the insecticidal function, two hemp varieties containing different levels of CBD at 3.2% and 0.8% in leaf tissues were used for the two-way feeding choice study. The test reveals that 3^rd^ instar *M*. *sexta* larvae showed clear preferences on the *Cannabis* leaf tissues containing 0.8% CBD over the 3.2% CBD tissues (Fig. 1A,B). When neonates were tested, they were unable to ingest any of the hemp tissues. Also, another feeding study shows that the high dosage of CBD (>1 mM) significantly inhibits the growth, mortality, and development of the insects, while the low dose (<1 mM) had no effect (Fig. 1C,D,E). Based on our data, we could not determine if the detrimental mechanism was due to neurotoxic effect or biochemical effect that CBD has on *M*. *sexta*’s digestive system. However, the feeding assay clearly demonstrated the preventive role of the CBD against pest insects. Indeed, numerous plants have been shown to exploit secondary metabolites (e.g., terpenes and phenolics) to protect plants from predators and microbial pathogens^28^. Additionally, it has been reported that high cannabinoid producing hemp varieties are more pest-resistant^6^ and also hypothesized that the cannabinoid level can be mediated by herbivory. It can be postulated that when CBD is highly accumulated in flowers, it is beneficial for the plants’ survival and reproduction. Despite the potential of CBD as a potent insecticide, in all likelihood, CBD use may not be feasible currently due to the high production and extraction costs. However, crude CBD-containing hemp extract of mature flowers may be used as a repellent in other crop species, as well as a companion crop^5,6^.

The latter feeding assay used MCT oil as a CBD solvent agent. To ensure that the inhibitory effects were not caused by the vehicle, another solvent agent, ethanol (200 proof) was used for dissolving CBD, and the feeding assay was repeated. Unexpectedly, greater than 1% EtOH in the diet appeared to be lethal for the caterpillars within 3 days. However, the larvae reared on the EtOH-containing AD with CBD display significantly lower mortalities. The CBD supplementation effectively rescued the insects from the EtOH stress, increasing the survival rate by up to 40% (Figs 1E and 4C).

To confirm the preventive role of CBD against EtOH-stress, another EtOH feeding study was implemented in a controlled-environment. The study confirmed that *M*. *sexta* larval growth and development were negatively correlated to the increase of EtOH in the diet. The chronic exposure to >1% EtOH significantly reduced the length, body weight, and survival rate. While *Drosophila* showed an increased mobility in the lower EtOH level^29^, the caterpillars used in this study did not show any increase in their mobility within sample subset of less than 1% EtOH. Additionally, the high dose of EtOH (>2%) significantly disrupted the food searching behavior (Fig. 3B).

Invertebrates have been used as a model system to investigate alcoholism and its social and behavioral effects due to the simplicity in anatomy, their sophisticated genetics, and molecular similarities to higher animals^30^. A series of EtOH feeding studies in honeybees demonstrated the negative influence in social communication and behaviors, a decrease in locomotion, and significant impairment of Pavlovian conditioning of proboscis extension^31^. Furthermore, a similar study shows that excessive ethanol intoxication by solutions containing ≥5% ethanol causes significant ethanol-induced stress in brain tissue that impairs honeybee behavior and associative learning^32^. Another EtOH study performed in *Drosophila* reveals the increase of locomotion at the low level of EtOH vapor, while higher doses cause reduced movement, loss of posture control, and immobility^30^.

Notably, *M*. *sexta* represents a lower EtOH tolerance when compared to mice that were able to survive at 36% EtOH ingestion^33^. The higher mortality is likely due to less alcohol-dehydrogenase (ADH) enzymes in insects than mammals that contain at least six medium-chain ADH classes (class I-VI)^34^. *Drosophila* produces only class III ADH enzyme, and the transcriptomic analysis performed on the *M*. *sexta* male moth antenna also revealed the presence of transcript encoding a class III-like ADH enzyme^35^. In addition, it is assumed that the respiratory system of *M*. *sexta* being mediated via spiracles caused the high casualty rate. The spiracles are external tracheal apertures located on each segment of the body for gas exchange, which may escalate the alcohol absorption into the insect system via the vapor.

The detoxifying mechanism of CBD against EtOH is still unknown. However, it is assumed that CBD effectively lowers the EtOH-induced oxidative stress, resulting in the increased growth, food consumption, mobility, and survival rate. Interestingly, CBD functions differently in the absence or presence of EtOH in the diet. In the absence of EtOH, >2 mM CBD became lethal to the insects (Fig. 1C,D,E). In contrast, the lethal doses show rescuing effects in the diets containing high EtOH concentrations (2%, 5%, and 10%). Our findings suggest that <1 mM CBD has no adverse effects on the insects’ growth, but a high dose of CBD (>2 mM CBD) can be inhibitory.

The chemical analysis indicates that CBD accumulation in the body of *M*. *sexta* was positively correlated to the amounts added to the diets (Fig. 4D). CBD was not detected from the control media (AD) itself and the larvae fed on AD. Also, CBD was not detected from the larvae reared on +10 uM CBD because it was under the detection limit. However, the larvae reared on AD + 1% EtOH + 100 uM CBD accumulates 0.0003% in the insect body, 0.0007% and 0.001% on +1 mM and +2 mM CBD diets, respectively. Only trace amounts were metabolized since the majority of CBD seemed to be excreted (Fig. 5C).

The electrophysiology data clearly show that CBD affects the signal transduction the ganglion axons, responded differently when treated EtOH and/or CBD. In Fig. 5C, two electric response peaks were observed, possibly the rapid first peak may have been caused by the myelinated axon and the delayed second peak by an unmyelinated axon. Based on the appearance of the first peak, EtOH caused a more rapid response compared to the larvae reared on AD and CBD-treated AD. Notably, the larvae treated with 1 mM CBD had delayed electric responses, but the magnitude of slope was much larger. Figure 5D presents the slope at different electric stimuli, revealing a significantly larger response in ganglia of CBD-treated larvae at all stimulus intensity after first stimulus at zero mA.

Cannabinoids, whether endogenous or exogenous, are potent regulators of neurotransmission^36^. THC and CBD bind to two distinct endocannabinoid receptors, CB1 and CB2, which belong the G-protein coupled receptor superfamily^37^. THC shows high affinity to CB1/CB2 receptors, which are primarily located in the brain and nervous system^37–39^. CBD has low affinity to both receptors, which is mostly found in peripheral organs and immune tissues^37,40^. Although CB1/2 receptors have been found in the nematode *Panagrellus redivivus* and several other invertebrates^41^, cannabinoid receptors have not been identified in insects to date^42^. Despite the absence of cannabinoid receptors in *M*. *sexta*, differential signal responses in the larval ganglia were evident between larvae reared on AD, AD + 1% EtOH, and AD + 1% EtOH + 1 mM CBD (Fig. 5C,D). The electrophysiology results suggest that the differential electric responses in the central nervous system (CNS) might have been caused by ‘non-CB receptors’ that are known to interact with cannabinoids in some species^43^. The non-CB receptors present in insects include 5-hydroxytryptamine (serotonin) receptors (5HT1A/2 A), transient receptor potential vanilloid cation channel receptors (TRPV), and nicotinic acetylcholine receptors (α7nACh) that are involved in a number of neurological and psychiatric responses^44^. In mammals, CBD has been found to act on these receptors. However, the binding affinity of CBD varies from species to species^43,45^.

The central nervous system is essential for signal transduction to modulate a variety of physiological responses including modulation of spiracles that are essential for respiration, gas and water exchange. This study’s findings demonstrate CBD’s role in modifying signal conductivity of the CNS. This modification may be the cause of the observed physiological changes, *i*.*e*., increased food searching activity, which would increase diet consumption, thus causing the differences in the length and weight observed in these caterpillars. All of these positive activities increased the survival rate of the EtOH-intoxicated *M*. *sexta*.

Here we report dual, contrasting roles of CBD in *M*. *sexta* larvae. The naturally occurring CBD in *Cannabis* primarily acts as a feeding deterrent against pests. Consequently, cannabidiol ingested effectively inhibits the larval growth and development, resulting in high mortality. Our results support a long-held belief for the defensive function of cannabinoids and would suggest the potential use of CBD-rich hemp extract as a repellent and/or as a companion crop. Secondly, lethal amounts of CBD function differently in the presence of EtOH stress, becoming protective. The CBD-administered larvae outperformed the larvae fed on the EtOH-only diet, significantly increasing survival rate by 40%. The electrophysiology results suggest that CBD affects the signal transduction of ventral ganglia in the CNS. This modification was beneficial to their growth and survival under chronic EtOH exposure. These findings could be a step forward to understanding the production of CBD as a part of defense mechanism in *Cannabis*, as well as the use of CBD as a non-traditional therapeutic treatment for alcohol abuse, addiction, and intoxication.

## Supplementary information

Supplementary Information

## Data Availability

The datasets that were generated and analyzed in the present study are available from the corresponding author upon reasonable request.
