# Supplementary material for: Contrasting Roles of Cannabidiol as an Insecticide and Rescuing Agent for Ethanol–induced Death in the Tobacco Hornworm Manduca sexta
Source: Sci Rep. 2019 Jul 19;9:10481. doi: 10.1038/s41598-019-47017-7 (PMC6642087; doi:10.1038/s41598-019-47017-7)
Supplement: Supplementary file 1 — Supplementary Information [file 41598_2019_47017_MOESM1_ESM.docx]

**Contrasting Roles of Cannabidiol as an Insecticide and Rescuing Agent for Ethanol–induced Death in the Tobacco Hornworm *Manduca sexta***

***Supplementary information***

**Sang-Hyuck Park^1,2^, S. Kyle Staples^1^, Eric L. Gostin^1^, Jeffrey P. Smith^1^, Jose J. Vigil^1^, Dustin Seifried^3^, Chad Kinney^2,3^, Christopher S. Pauli^2^, and Brian D. Vanden Heuvel^1*^**

^1^Department of Biology, ^2^Institute of Cannabis Research, and ^3^Department of Chemistry, Colorado State University-Pueblo, Pueblo CO 81001, USA.

^*^Correspondence and requests for materials should be addressed to B.V.H. (email: brian.vandenheuvel@csupueblo.edu)

1. **Supplementary Table**

**Table 1**. The total CBD and THC amounts of *C. sativa* L. hemp varieties (Colorado Gold and Green Thunder) used in this study.

|  | *C. sativa* L. hemp  (Colorado Gold) | *C. sativa* L. hemp  (Green Thunder) |
| --- | --- | --- |
| Total CBD | 0.81% | 3.2% |
| Total THC | 0.09% | 0.11% |

A total THC and CBD were calculated by using the formula; % of CBD _total_ = % of CBD + (% of CBDA × *0.877) and % of THC _total_ = % of THC + (% of THCA × 0.877). *0.877 is a molecular weight ratio of CBDA/CBD and THCA/THC.

1. **Supplementary Figure**


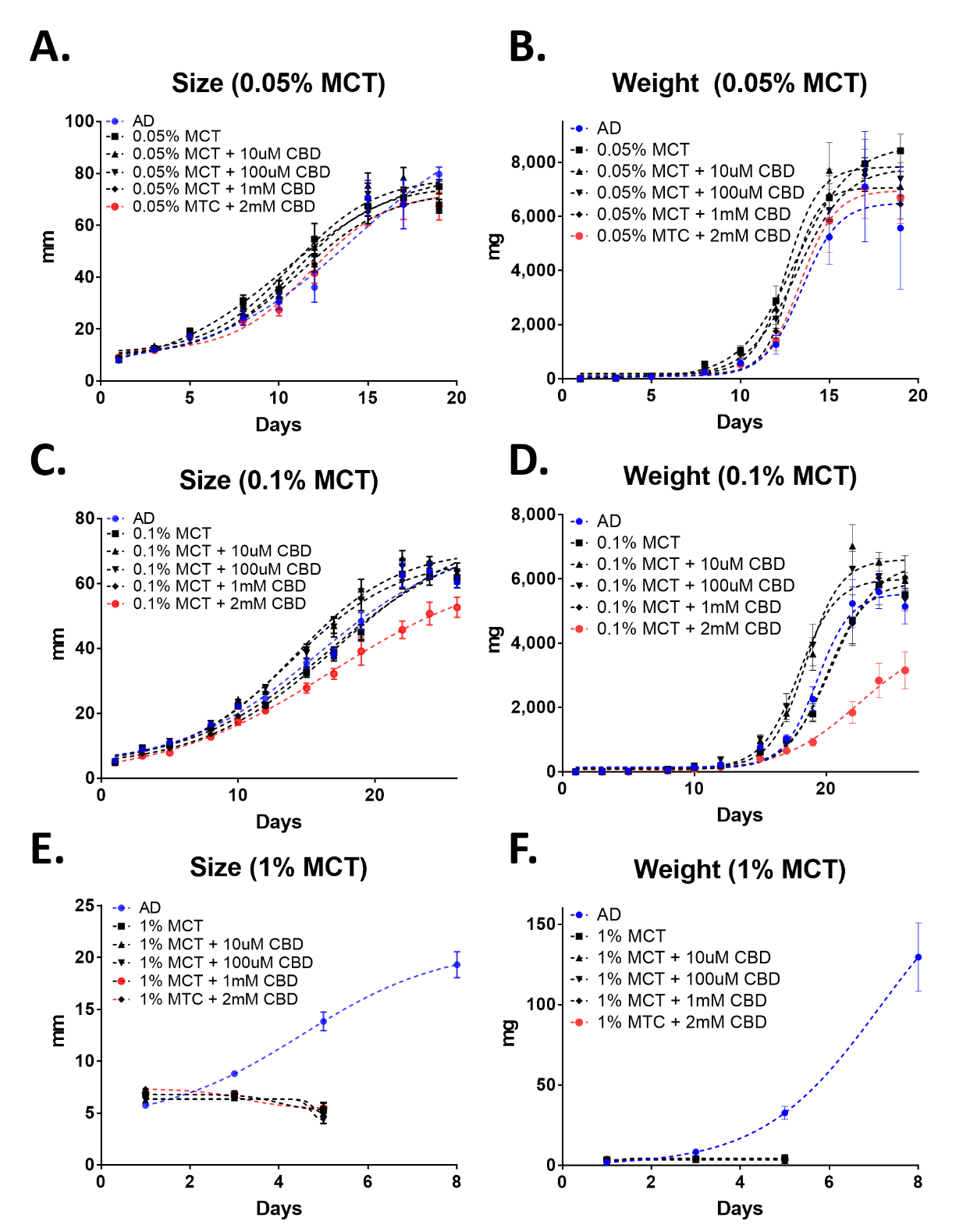


**Figure 1**. The effects of medium-chain triglyceride (MCT) on the growth of tobacco hornworm *Manduca sexta* (n=20-22). First instar larvae were grown on artificial diet (AD) containing various amounts of CBD dissolved in 0.05% (A, B), 0.1% (C, D), and 1% MCT oil (E, F).
